# Examining suicidality in relation to the menopause: A systematic review

**DOI:** 10.1371/journal.pmen.0000161

**Published:** 2024-11-13

**Authors:** Nayra A. Martin-Key, Erin L. Funnell, Eleanor J. Barker, Sabine Bahn

**Affiliations:** 1 Cambridge Centre for Neuropsychiatric Research, Department of Chemical Engineering and Biotechnology, University of Cambridge, Cambridge, United Kingdom; 2 University of Cambridge Medical Library, University of Cambridge, Cambridge, United Kingdom; CHINTA Research Bangladesh, BANGLADESH

## Abstract

Suicide is one of the leading causes of deaths worldwide, with an estimated 1 in 100 deaths being attributable to suicide. Whilst rates of suicide are higher in men, evidence suggests that suicide attempts are more frequent in women. Suicidality data indicates that deaths by suicide in women are highest in those in midlife, warranting investigation into the relationship between the menopause and suicidality. The current study aimed to review the existing literature examining the relationship between suicidality and the menopause using a systematic review approach. A systematic literature search of MEDLINE, Cochrane Library, Scopus Web of Science, PsycINFO, and Embase databases was conducted in October 2023. Two authors independently screened the titles and abstracts of identified articles against the eligibility criteria. Any inconsistencies were discussed and resolved. This process was subsequently repeated with the articles’ full-text. Risk of bias was assessed using the Quality Assessment Tool for Studies with Diverse Designs (QATSDD). Relevant data were extracted and summarised in both a tabulated and narrative form. A total of 28 studies met the inclusion criteria, with the findings revealing a complex relationship between the menopause and suicidality. Several studies highlighted that the perimenopause period shows a higher prevalence of suicidal thoughts compared to pre-menopausal and post-menopausal stages. Conversely, some studies indicated increased suicidality during the post-menopausal phase, while others noted elevated suicidality in pre-menopausal individuals and those with primary ovarian insufficiency. Critically, several studies found no link between hormonal status and suicidality. The quality of the studies also varied, with a lack of involvement from individuals with relevant lived experience being a consistent methodological flaw across all the included studies. Overall, the current evidence on menopause and suicidality is mixed. Further research is needed to unravel the relationship between menopause and suicidality.

## Introduction

Suicide is one of the leading causes of death worldwide, with an estimated 1 in 100 deaths being attributable to suicide [1]. Whilst rates of death by suicide are higher in men, evidence suggests that suicide attempts are more frequent in women [2]. There has been increasing research investigating suicide in the midlife period [3], with global suicidality data indicating that risk of death by suicide increases with advancing age [4], and in some countries, the highest suicide mortality rate for women appears to occurs in midlife [5, 6].

The menopause, typically occurring in midlife, represents a period of substantial life changes, characterised by the decline in ovarian functioning in natural menopause or initiated by certain surgeries or treatments in the case of medically-induced menopause. The menopause comprises a constellation of vasomotor, sleep, physical, sexual and psychosocial symptoms, and whilst the individual menopause experience is highly unique, the impact of menopausal symptoms on health-related quality of life [7] and wellbeing can be extremely challenging. The menopause also appears to be associated with depressive symptoms [8], with it being increasingly recommended that depression screening is integrated into standard menopausal care [9].

Indeed, the menopause transition and post-menopause appear to increase the risk of depressive symptoms [10]. The link between depressive symptoms and suicidality is well documented [11–13], with suicidality listed as a diagnostic criterion of major depressive disorder (MDD) [14]. Critically, depressive symptoms differ between premenopausal and menopausal individuals, with menopause-related depression being reportedly more so characterised by irritability, sleep complaints, and fatigue [15]. Given evidence that irritability is associated with increased suicidality in depressed individuals [16], it is possible that some menopause-related depressive symptoms may be strong candidates for screening to identify heightened risk of suicidality in this population.

Additionally, anxiety symptoms, which are reportedly common in the menopause [17] with a potentially increased susceptibility in this period [18], have been revealed to be associated with suicidal ideation [19–21]. Moreover, additional menopausal experiences may influence the risk of suicidality, such as feelings of hopelessness, which has previously been implicated in suicidality [22], with feeling depressed and feeling hopeless both identified as highly important risk factors in suicidality [22]. Hopelessness is reported by menopausal women who perceive not receiving appropriate intervention to manage symptoms [23], with menopausal women reporting fear that symptoms will persist or worsen [24].

Understanding the clinical profile of suicidality in relation to the menopause is crucial for effective screening and management of such symptoms in this population, as well as to identify strategies to address the associated risks. Given the importance of this research area, we aimed to conduct a systematic review examining available literature investigating suicidality (including suicidal ideation, suicide risk, suicide attempts, and death by suicide) in the menopause. The findings of the current literature review have the potential to inform the understanding of the clinical profile of menopausal suicidality by synthesising current evidence, in addition to identifying gaps in the research landscape, which is crucial for shaping research priorities.

## Methods

### Overview

The current literature review has been registered with the International Prospective Register of Systematic Reviews (PROSPERO; https://www.crd.york.ac.uk/PROSPERO/display_record.php?RecordID=478572). The protocol was developed using the Preferred Reporting Items for Systematic Review and Meta-Analysis Protocols (PRISMA-P; [25]) recommendations. Several amendments were made to the original protocol (please see the Prospero record for details); broadly, the inclusion criteria were narrowed to only include articles published in English due to translation limitations encountered after searches were completed. Additionally, as the search terms used were broad, studies that only examined factors *associated* with suicidality in peri or post-menopausal individuals (e.g., relationship status, physical health status, psychiatric history, treatment effects), rather than menopausal status itself, were excluded. The PRISMA checklist for the current study is available in S1 Table.

### Eligibility criteria

We conducted a systematic review of literature focused on suicidality (including suicidal suicide ideation, suicide risk, suicide attempts, and death by suicide) in the menopause and menopause transition. As such, studies of suicidality not focused on individuals in the perimenopause, post-menopause, or medically-induced menopause were excluded. Studies examining the association between treatment or management strategies for the menopause or menopausal symptoms (e.g., hormone replacement therapy) and suicidality were also excluded. Additionally, studies investigating factors associated with suicidality in peri or post-menopausal individuals which were not specifically related to the menopause (e.g., relationship status, physical health status, psychiatric history) were excluded.

The population of interest included individuals in the perimenopause (also known as the menopause transition), post-menopause (also known as natural menopause), and medically-induced menopause (i.e., menopause resulting from medical treatment including surgery). Participants of any gender, age, ethnicity, and geographical location were included.

Any study design was considered for inclusion in the current literature review, aside from systematic reviews, meta-analyses, and opinion pieces. There was no limit on the date of publication. Only articles published in English were considered.

### Search strategy

The search terms used in the current study were defined using the SPIDER (Sample, Phenomenon of Interest, Design, Evaluation, Research type) tool (Table 1). The SPIDER tool was chosen for the current systematic review as it is suitable for searching for qualitative and mixed-methods publications, as well as quantitative publications [26]. The following databases were searched: MEDLINE, Cochrane Library, Scopus Web of Science, PsycINFO, and Embase. Searches were completed on October 25, 2023. Grey literature relevant to the focus of the current study (e.g., clinical trial databases, unpublished theses, reports, conference presentations, guidance or reports produced by medical bodies or charities, news articles) were also identified by hand. Other potentially eligible trials or publications were identified by hand searching the reference lists of retrieved publications, and included studies in any identified systematic reviews, meta-analyses, and opinion pieces. Hand searches were completed on December 8, 2023.

**Table 1:** The SPIDER framework as implemented for the current systematic review.

Sample	Menopausal individuals (including the perimenopause, post-menopause, and medically-induced menopause)
Phenomenon of Interest	Suicidality (including suicidal ideation, risk, attempts, and completions)
Design	Published literature of any research design, grey literature
Evaluation	Prevalence, clinical presentation or characteristics, views, experiences
Research type	Published qualitative, quantitative, and mixed-methods studies. Grey literature including but not limited to unpublished theses, reports, conference presentations, guidance, or reports produced by medical bodies or charities, and news articles

Keywords and subject headings related to the topic and population of interest were identified in a preliminary scan of the literature and chosen in consultation with a medical librarian (EB). Due to the broad scope of the current systematic review, the design, evaluation, and research type components of the SPIDER tool were not included in the search terms to avoid inadvertently excluding relevant articles (see Table 2).

**Table 2:** Search terms.

Component of the SPIDER framework	Related search terms
Sample	menopaus* OR perimenopaus* OR peri menopaus* OR peri-menopaus* OR postmenopaus* OR post menopaus* OR post-menopaus* OR climacteric
Phenomenon of Interest	suicid* OR self killing OR self immolation OR selfimmolation

### Screening and article selection

**Fig 1:**
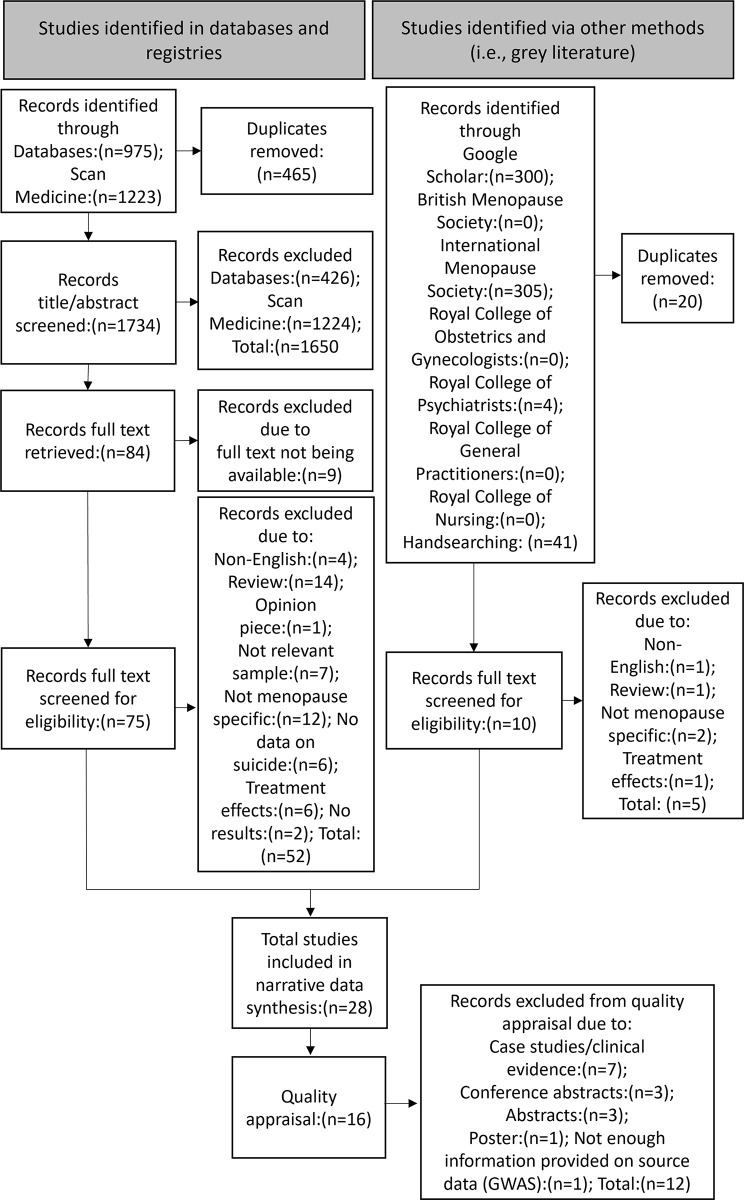
PRISMA flowchart showing inclusion and exclusion decisions.

All articles identified from the database searches were entered into the systematic review software Rayyan. Any duplicate results from the database searches were removed from the article dataset. Two authors (EF and NMK) independently screened the titles and abstracts of the identified articles under blinded conditions. To decide whether an article’s full text should be further screened, the article’s title and abstract were evaluated for eligibility against the inclusion criteria and then labelled as “exclude,” “include,” or “maybe.” For an article to be included, both reviewing authors had to label it as “include.” Articles were excluded if both of the reviewing authors labelled it as “exclude”. Articles labelled as “maybe” or any disagreements were discussed between the reviewing authors following unblinding until a consensus was reached. Following this, the full texts of the “included” articles were then further screened by the two reviewing authors independently to determine final eligibility. Again, any disagreements in the inclusion decisions of the articles based on the full text were discussed until a consensus was reached. Reasons for full-text exclusions were recorded (Fig 1).

### Data extraction

Two authors (EF and NMK) independently examined the full-texts of the final dataset of included papers to extract relevant data for the current literature review. Extracted data included (1) publication details: author(s) and date; (2) study design and methodology: geographical location, study design, sample characteristics (e.g., sample type, sample size, sample age, ethnicity, education), (3) measures of interest: measure of menopause status, measure of suicidality, (4) outcomes: main findings. All extracted data were entered into a standardised Excel spreadsheet for storage. Disagreements in data extraction were resolved through discussion following unblinding.

### Quality appraisal

To assess the quality of included studies, the Quality Assessment Tool for Studies with Diverse Designs (QATSDD; [27]) was utilised. The QATSDD is a 16-item quality assessment tool, with a scoring scale from 0 = “Not at all” to 3 = “Complete”. Included quantitative studies, were assessed against the relevant 14 QATSDD items. Included qualitative studies were assessed against the relevant 13 QATSDD items. The overall percentage QATSDD score for each included study was calculated using the score of the study divided by the total possible score for the study type (i.e., 14 for quantitative studies and 13 for qualitative studies).Two reviewing authors (EF and NMK) independently assessed the included articles using the QATSDD and entered scores into a standardised Excel spreadsheet. Disagreements in quality appraisal were resolved through discussion.

### Data analysis and synthesis

Extracted data were summarised in a tabulated form. Additionally, a narrative summary of the included articles was produced. Only data specific to the menopause in relation to suicide was extracted. In cases where specific data (e.g., sample characteristics) for eligible studies could not be obtained, the study was still included in the review. A description of the missing data for each study was provided in the relevant section of the results.

## Results

### Included studies

In total, 2,096 articles were retrieved, of which 85 (4.06%) were identified for full-text review (S2 Table). Of these, 28 (32.94%) met the criteria for inclusion (24 full-texts, 3 conference abstracts, and 1 poster; see Fig 1 and Table 3). Notably, a large portion of the studies failed to meet the inclusion criteria, primarily because risk factors for suicidality could not be specifically attributed to the menopause. Other exclusions regarded studies being reviews and there being no data on suicidality. For an overview of the study characteristics (i.e., study design, publication dates, and country) and key relevant results of the 28 included studies see Table 3. For complete details of the 28 included studies see S3 Table.

**Table 3:** Summary of included studies. For the complete table including sample characteristics, details of measures, and all findings relevant to the review see S2 Table.

Ref	Author	Year	Country	Study design	Main findings
**28**	An et al.	2022	South Korea	Cross-sectional	Menopause increased the risk of suicidal ideation, when comparing the early menopause transition (OR = 1.3, 95% CI = 1.2–1.5, *p* < 0.001), late menopause transition (OR = 1.2, 95% CI = 1.0–1.3, *p* < 0.001), and post-menopause (OR = 1.6, 95% CI = 1.4–1.9, *p* < 0.001), against the pre-menopause. After adjusting for sleep duration and sleep quality, only the association with suicidal ideation with the early menopause transition and the post-menopause stages remained statistically significant. The association between the early menopause transition and the post-menopause remained similar even when adjusting for history of suicidal behaviour. The post-menopause stage was associated with the highest likelihood of either isolated suicidal ideation (early menopause transition: OR = 1.2, 95% CI = 1.0–1.4; late menopause transition: OR = 1.0, 95% CI = 0.8–1.2; post-menopause: OR = 1.3, 95% CI = 1.1–1.6) and concurrent depression and suicidal ideation (early menopause transition: OR = 1.4, 95% CI = 1.2–1.6; late menopause transition: OR = 1.2, 95% CI = 1.0–1.5; post-menopause: OR = 1.8, 95% CI = 1.5–2.3), when compared to premenopause.
**29**	Baca-Garcia et al.	2003	Spain	Cross-sectional	The L genotype frequency was elevated in menopausal suicide attempters (38%) compared to fertile suicide attempters (16%) (χ2 = 5.3, df = 1, *p* = 0.002). In non-menstrual phases with higher hormone levels, menopausal suicide attempters showed a higher L genotype frequency (38%) than fertile suicide attempters (13%) (χ2 = 7.7, df = 1, *p* = 0.005). However, it did not significantly differ from the L genotype frequency in fertile suicide attempters during menses (27%) (χ2 = 2.4, df = 1, p = 0.12)
**30**	Baca-Garcia et al.	2010	Spain	Cross-sectional	Suicide attempters experiencing menopause or amenorrhea, characterised by low estradiol/low progesterone states, exhibited elevated suicidal intent (SIS score >14) at rates comparable to menstruating women (menopause: 45%; amenorrhea: 31%; χ2 = 1.7, df = 2; *p* = 0.415), but significantly higher than during other menstrual cycle phases (χ2 = 11.6, df = 2; *p* = 0.003)
**31**	Fleming & Warneboldt	1990	Canada	Case study	"Mrs. A., a 68-year-old married woman. […] The patient’s complaint was "bad nerves" associated with chronic depression, which began at the time of her menopause, had persisted for the last 20 years […]. Although not actively suicidal, she remarked that her life, as she was currently experiencing it, was not worth living. […] Her medical history included a nearly fatal suicide attempt, 15 years earlier […]"
**32**	Fujikane et al.	2023	Not specified	Genome-wide association study	Suicide attempts were significantly genetically correlated with an earlier age at menopause (−0.4 ± 0.1, *p* = 1.3 × 10^−8^). This correlation remained statistically significant after adjusting for mental disorders (−0.5 ± 0.2, *p* = 1.0 × 10^−3^).
**33**	González-Rodríguez et al.	2013	Spain	Cross-sectional	No differences were found in suicidal behaviour between the two groups (premenopausal and postmenopausal; no statistics provided).
**34**	González-Rodríguez et al.	2015a	Spain	Cross-sectional	Age at start of the menopause was positive correlated with the severity of suicidal ideation (r = 0.5, p = 0.038). However, when controlling for social support (e.g., number of family members including children), age at the onset of the menopause was no longer significantly associated with severity of suicidal ideation.
**35**	González-Rodríguez et al.	2015b	Spain	Cross-sectional	There were no significant differences observed in lifetime suicidal ideation and behaviour between premenopausal (n = 5, 20.0%) and post-menopausal (n = 9, 16.4%) onset of a delusional disorder (χ2 = 0.16, df = 1, *p* = 0.755) or in suicidal ideation intensity (premenopausal = 3.1 (SD = 6.53); post-menopausal = 3.5 (SD = 7.4); t = -1.2, df = 71, *p* = 0.172).
**36**	González-Rodríguez et al.	2016	Spain	Cross-sectional (poster)	No significant differences were found among the three groups (men, pre-menopausal women, post-menopausal women) in the proportion of lifetime suicidal ideation (X2 = 1.7(2), *p* = 0.423; men: n = 33, 45.8%; pre-menopausal: n = 16, 51.6%; post-menopausal: n = 24, 38.1%). Similarly, there were no significant differences in the proportion of lifetime suicidal behaviour (X2 = 0.6(2), *p* = 0.716; men: n = 19, 25.7%; pre-menopausal: n = 8, 25%; post-menopausal: n = 16, 25.4%). When adjusting for confounders, there were significant differences in current suicidal ideation intensity among the three groups (*p* = 0.039), with the post-menopausal group scoring higher than the other two. Significant differences were also observed in the impact of deterrents on suicide attempts between the three groups (*p* = 0.004), with the post-menopausal group less likely to be deterred compared to the others. While there were no significant differences in reasons for suicidal ideation (i.e., to gain attention vs. to stop the pain; *p* = 0.062), the data suggests a trend toward the post-menopausal group being more likely to attempt suicide to stop the pain relative to the other groups.
**37**	Hemphill & Reiss	1944	UK	Case study	"A woman aged 41 was admitted to the Bristol Mental Hospital on Dec. 21, 1940. […] There was no history of antecedent mental or physical illness. The menses ceased abruptly at the age of 39 without hot flushes or other subjective menopausal disturbances. About six months before admission she became depressed, lost appetite, and imagined that her viscera were deranged and the bowels "stopped up"; she attempted suicide. This was the mental condition on admission. […] Menses have not returned, and the vaginal smear, as before treatment, is of the post-menopausal atrophic type"
**38**	Høyer & Lund	1993	Norway	Longitudinal, retrospective	The suicide mortality rates were highest in the age group 35 to 44 years (i.e., premenopausal) regardless of marital status and parity, with a strong decline in suicide mortality rates with increasing age. Postmenopausal women had a larger decrease in suicide mortality than premenopausal women, with increasing age.
**39**	Incalzi et al.	1989	Italy	Case study	**"**A 45 year old woman, experienced progressive deterioration of memory, loss of concentration, anxiety, tiredness, insomnia with nightmares and arousals characterized by unexplained fear and, rarely, thoughts of suicide. She developed a profound depression, so that 2 months later in November 1987 she decided to discontinue her job in the stock market. A diagnosis of menopausal-related anxious depressive syndrome was made by her general practitioner and confirmed by two psychiatrists."
**40**	Jones	1900	Republic of Ireland	Clinical observation	"I have myself known two cases [of suicide] occurring during the menopause”
**41**	Jung et al.	2019	South Korea	Cross-sectional	There was no significant difference between the two groups in terms of one-year suicidal thoughts (menstruation = 9.2%, early menopause = 9.7%), as well as one-year suicidal attempts (menstruation = 1.5%, early menopause = 1.5%), and suicidal thought rate (menstruation = 9.2%, early menopause = 9.7%).
**42**	Kornstein et al.	2010	US	Cross-sectional	Premenopausal and perimenopausal women were approximately twice as likely to report a past suicide attempt than were post-menopausal women (premenopausal, 23%; perimenopausal, 20%; postmenopausal, 12%; *p* < 0.0001). After adjustment for baseline differences, group differences were found for suicidal ideation (OR Peri vs Pre, 1.2 [0.9–1.5]; OR Post vs Pre, 1.5 [1.2–2.1]; *p* = 0.007). Post-menopausal women were more likely to have suicidal ideation than premenopausal women, and perimenopausal women were less likely to have suicidal ideation than were post-menopausal women.
**43**	Lipsitz et al.	2021	Canada	Longitudinal, prospective	There was no significant difference in suicidal ideation at baseline (i.e., pre-treatment with intravenous ketamine): pre-menopause = 1.9 (1.0) vs. post-menopause = 1.4 (1.1) (*p* = 0.440) in women with treatment resistant depression.
**44**	Little & Cameron	1937	Canada	Case study	Case 5: “A 66-year old widow. Diagnosis, involutional melancholia. […] One or two feeble attempts at suicide had been made. […] Her previous history showed two previous breakdowns similar to the present one, the first following the menopause at 48 years and the second at 53 years. […] Her personality prior to the menopause had been stable.” Case 6: “A 60-year old widow, diagnosed as a manic-depressive (depression). […] She became depressed and agitated, felt hopeless, and expressed suicidal ideas. […] The family history showed a sister to have had a mental breakdown at the menopause. […] The menopause occurred at 46.” Case 8: “A 50-year old married woman. Diagnosis, depression at the involutional period. Her illness had begun four years before with fatigue and inability to do her work. A few months later her father died, and about the same time menopausal changes appeared. […] Six months later headaches commenced. Vague anxiety and a feeling of tenseness came on, she slept poorly, and had difficulty in concentrating. Her condition remained practically unchanged, except for mild suicidal thoughts prior to admission.” Case 11: “Patient is a 53-year old married woman. Diagnosis, manic-depressive psychosis, depressed type with agitation. […] She expressed suicidal ideas, but did not act upon them. Her family and personal history were negative. Her previous personality was quite stable. The menopause occurred a little over a year ago and completed nine months ago.”
**45**	Zhiqiang et al.	2016	China	Case study	"The patient had undergone a subtotal hysterectomy because of uterine leiomyoma three years prior at a local hospital. [. . .] A cervical biopsy was implemented after admission and a pathology examination revealed cervical leiomyosarcoma. On January 17, 2013, the patient underwent a radical resection of the cervix, bilateral salpingo-oophorectomy, and pelvic lymphadenectomy. [. . .] The patient suffered from severe menopausal symptoms after gynecological surgery and received hormone replacement therapy. [. . .] The mental state of the patient had been very poor due to menopausal symptoms and from learning that she was suffering from malignant tumor. She finally committed suicide by jumping off a building on April 21, 2013."
**46**	Moseley et al.	2020	UK	Focus group study	Participants with autism commented on suicidality in relation to the menopause: “Mental health issues at menopause could impact on suicidal tendencies”; “Yes indeed and I think that the extra stress all the above brings can result in feeling too tired and overwhelmed to carry on… It’s like, your everyday life, just to appear ’normal’ is bad enough, but when M happens, it all gets too much… I wonder if the low life expectancy for autistic women is linked to menopause because it all gets too much.”
**47**	Murphy et al.	2013	Qatar	Focus group study	In the context of menopause, some women in focus groups perceived religion as establishing strong social networks, preventing potentially depressive outcomes like suicide: ". . .our religion makes us united and close and prevents us from suicide. . ."
**48**	Nakanishi et al.	2023	Japan	Longitudinal, prospective	A binomial regression analysis of suicidal ideation at follow-up was conducted with premenopause as a reference. After adjusting for baseline suicidal ideation, history of depression, physical factors, health-related behaviours, social class, and psychosocial factors, perimenopause after baseline was significantly associated with a greater likelihood of suicidal ideation at the follow-up (OR = 1.9, C I = 1.3–3.0, *p* = 0.003). A Cox proportional hazards survival regression analysis revealed a higher hazard ratio (HR) for suicidal ideation at follow-up in individuals in the perimenopause (HR = 1.6, 95% CI = 1.0–2.4, *p* = 0.045). When menopausal stage was treated as a time-varying variable, Cox regression analysis revealed that postmenopausal status was significantly associated with a lower hazard ratio of suicidal ideation across the study stages (HR = 1.0, 95% CI = 0.97–1.0, *p* = 0.008).
**49**	Patel et al.	2016	US	Cross-sectional	Based on the MINI, 34.5% of the sample had suicidal ideation. Compared to pre-menopausal women, post-menopausal women experienced a higher prevalence of suicidal ideation (39% vs. 31%, *p* value not reported)
**50**	Rocha et al.	2019	Brazil	Cross-sectional	10.5% of the sample endorsed suicidal ideation. Among these, 42.5% were between the ages of 52 and 65 years old. There was a statistically significant association between suicidal ideation and mood disorder (*p* = 0.000), symptoms of climacteric (*p* = 0.000), sleep (*p* = 0.000) and age at first childbirth (*p* = 0.021).
**51**	Ryu et al.	2022	South Korea	Cross-sectional	There was a significant difference in the PHQ-9 suicide item between the three groups (primary ovarian insufficiency (POI), early menopause, menopause after the age of 45; *p* = 0.016; POI: mean = 0.3, SD = 0.5; early menopause: mean = 0.1, SD = 0.3; menopause after 45 years: mean = 0.1, SD = 0.3), with the POI group scoring significantly higher than the menopause after 45 years group. Women with POI had a significantly higher odds of suicidal ideation than those who experienced menopause after 45 years of age; this association remained significant in 2 multivariable regression models after the adjustment for several confounding factors: model 2 was adjusted for age (OR = 5.9; 95% CI = = 1.5–23.8), and model 3 was further adjusted for BMI, and education, household income, and walking levels (OR = 4.2; 95% CI = 1.0–17.7)
**52**	Sherr et al.	2016	UK	Cross-sectional	There was no significant difference in the distribution of suicidal ideation between the two groups (premenopausal and postmenopausal; *p* = 0.850), although a higher proportion of the post-menopausal group (n = 29, 56.9%) endorsed suicidal thoughts over the last week relative to the pre-menopausal group (n = 33, 54.1%). When examining mental health comorbidity, a trend was observed among the post-menopausal group, indicating a lower percentage without any mental health challenges (26.1% compared to 42.4%). There was a higher prevalence of multiple comorbidities in the post-menopausal group, involving two or three conditions such as anxiety, depression, and suicidal ideation (26.9% compared to 8.5%). However, this difference did not reach statistical significance (χ2(1) = 7.0, *p* = 0.070).
**53**	Usall et al.	2009	Belgium, France, Germany, Italy, the Netherlands, and Spain	Cross-sectional	Peri-menopausal women exhibited a sevenfold higher prevalence of suicide ideation (7.8%) compared to other groups (1.1% for pre-menopause and 1.0% for post-menopause; *p* = 0.004). Suicide ideation prevalence for men was similar across groups (ranging from 0.6% in the older group to 1.0% for those aged 46 to 57 years; *p* = 0.770). Compared to pre (0.5%) and post-menopausal (0.6%) women without mental disorders, peri-menopausal women (8.2%) showed a higher prevalence of suicide ideation (p value not reported). In comparison to pre (5.9%) and post-menopausal women (4.1%) with 12-month mood disorders, peri-menopausal women (14.2%) displayed a higher prevalence of suicide ideation (p value not reported. The association of suicide ideation with peri-menopause remained significant after adjusting for mental disorders and sociodemographic (OR = 9.0; 95% CI = 1.9–43.3 compared to pre-menopausal women; OR = 6.67; 95% CI = 1.3–33.3 compared to post-menopausal women). Conversely, the oldest men showed decreased odds for presenting suicide ideation (OR = 0.02, 95% CI = 0.0–0.7)
**54**	Weiss et al.	2016	US	Longitudinal, prospective	After adjusting for age, physical health, and hormonal status, women at high suicide risk (n = 53) had a mean anxiety score of 12.4 (SD = 5.1), compared to the low-risk group (n = 245) with a mean score of 9.99 (SD = 6.5). The model explained 11% of the variance in suicide risk. Although hormonal status did not significantly predict suicide ideation or attempts (*p* = 0.741), it is noteworthy that 33% (6/18) of women who had undergone surgical removal of the ovaries or uterus were in the high-risk group, while no pregnant woman was. Suicide risk varied across hormonal classifications, ranging from 12% of perimenopausal women to 18.5% of premenopausal women with regular menstruation reporting elevated risk
**55**	Worsley et al.	2016	Australia	Case study	"A highly functional 53-year-old woman with no psychiatric history presented with abrupt onset anxious and depressive symptoms, culminating in two high lethality suicide attempts. Her depression stabilised somewhat on high dose venlafaxine 225mg and olanzapine 5mg. However, she improved significantly after her general physician (GP) prescribed tibolone hormone replacement therapy (HRT), in response to the emergence of physical menopausal symptoms of hot flushes and sweating"

Key. OR: Odds ratio; CI: Confidence interval; HR: Hazard ratio

### Study and sample characteristics

The majority of the studies included participants with psychiatric conditions [32–36, 39, 42–44, 46, 54, 55]. Three studies recruited participants via emergency care settings [29, 30, 48]. Three studies included participants with other physical health conditions [37, 45, 52]. Two studies included participants with neurological conditions [31, 49]. Finally, one (clinical observation) study described the population studied as “menopausal women” but did not provide further details [40].

Sample sizes ranged from one [31, 37, 39, 45, 55] to 989,949 [38]. Participant ages ranged from 18 to > 90 years, though not all studies provided this information. 23 studies did not provide information on ethnicity [28, 30–41, 43–48, 50, 51, 53, 55]. Of the remaining five studies, four of these primarily included Caucasian participants [29, 42, 49, 54], whilst one study had a higher proportion of Black participants [52].

### Measure of menopausal status

Nine studies relied *solely* on self-reported data to establish menopausal status [28, 33–36, 46, 53–55]. Of these, one study [28] grouped participants into four menopausal stages (pre-menopause, early menopause transition, late menopause transition, and post-menopause) based on the Stage of Reproductive Aging Workshop + 10 criteria [56]. Two studies [35, 36] employed the definition of the menopause put forward by the International Menopause Society guidelines [57] to categorise participants. Another provided clear definitions of the pre-menopause, perimenopause, and the post-menopause [53]. A further study categorised participants into six hormonal status groups using six self-report items but did not provide clear definitions for all of the groups [54]. Four studies asked participants about their hormonal status but it was unclear whether they had provided definitions for these groupings [33, 34, 46, 55].

Three studies employed age as a proxy to establish hormonal status [38, 43, 52], with these studies defining participants aged 44 or below as pre-menopausal and participants aged 45 and over as post-menopausal.

One case study [45] assessed hormone levels in the blood to establish menopausal status. Three studies [29, 30, 50] utilised a combination of hormone levels in the blood and self-reported data to establish hormonal status. For the latter three studies, it was unclear whether definitions were provided for hormonal status groupings.

Three studies [42, 48, 51] employed a combination of self-reported data and age as a proxy for hormonal status to categorise participants, with one study [51] defining menopause as the absence of menstruation for over 12 months, but not providing a definition for primary ovarian insufficiency (POI) and early menopause, utilising age brackets instead. The remaining two studies did not appear to provide clear definitions for their hormonal groupings [42, 48].

Finally, one case study employed a vaginal smear to determine hormonal status [37].

The remaining eight studies did not provide a description of the specific measure of menopausal status employed [32, 39, 40, 41, 44, 49].

### Measure of suicidality

#### Suicidal ideation or thoughts

Overall, 16 studies had data investigating the interaction between suicidal ideation/thoughts and the menopause.

The most commonly used validated measure was the Columbia-Suicide Severity Rating Scale [58], which was used in four studies [33–36], Two studies [43, 54] included the suicidal ideation item of the 16-item Quick Inventory of Depressive Symptomatology (QIDS-SR16 [59]). Other measures included: the suicidal ideation item of the 30-Item Inventory of Depressive Symptomatology-Clinician Rated (IDSC-30 [60]) [41], the 4-item subscale of the 28-item Japanese version of the General Health Questionnaire (GHQ [61]) [48], the Korean version of the 9-item Patient Health Questionnaire (PHQ-9 [62]) [50], Beck’s Suicidal Intent Scale (SIS [63]) [30], using a cut-off score of 14 to categorise participants into low vs. high intent, as has been used in previous research [64], and the suicidality subscale of the Mini International Neuropsychiatry Interview (MINI [65]) [49].

Five studies employed author-generated measures of suicidal ideation/thoughts using questions that had not been previously validated [28, 41, 50, 52, 53]. Of these, two studies used binary questions to examine suicidal ideation/thoughts [28, 53] The remaining three studies did not provide details regarding the questions that had been used [41, 50, 52].

#### Suicidal attempts

Five studies examined the relationship between suicidal attempts and the menopause.

One study [29] measured suicidality within 24 hours of a suicide attempt, using the definitions put forward by the US National Institute of Health criteria [66].

One study [35] used the Columbia-Suicide Severity Rating Scale [58] to assess suicidal behaviour (i.e., previous attempts).

Two studies [41, 42] asked about previous suicide attempts, but the specific questions asked to assess this were not reported.

A Genome-Wide Associate Study (GWAS) study [32] examined suicide attempts but did not provide information regarding how this was measured.

#### Suicidal mortality

One study [38] investigated deaths from suicide according to the International Classification of Diseases (8^th^ Revision; [67]) as was stated on participants’ death certificate.

#### Other studies of suicidality

Of the seven case studies/clinical observations, five used self-reported information (i.e., about suicidal thoughts, ideation, attempts) disclosed in a clinical setting [31, 37, 39, 44, 55]. Two case studies [40, 45] reported information on death by suicide.

A measure of suicidality was not relevant for the two qualitative studies [46, 47].

### Key findings

#### Suicidal ideation or thoughts

Overall, 16 studies included data investigating the interaction between suicidal ideation/thoughts and the menopause. The evidence was mixed, with six studies presenting findings that there was no difference in suicidal ideation/thoughts based on menopausal status [33–35, 41, 43, 54]. In contrast, 10 studies presented findings of an association between the menopause and suicidal ideation/thoughts [28, 30, 36, 42, 48–53]. Of these, two did not investigate a specific menopause stage [30, 50], five identified a higher incidence of suicidal ideation/thoughts in the post-menopause stage [28, 36, 49, 42, 52], two identified a higher incidence of suicidal ideation/thoughts in the peri-menopause stage [48, 53], and one identified a higher incidence of suicidal ideation/thoughts in individuals with early onset (i.e., <40 years) menopause [51].

#### Suicide attempts

Five studies presented data on suicide attempts related to the menopause. Once again, the evidence was mixed, with two studies finding no association between the menopause and suicidal attempts [35, 41]. On the other hand, one study found that both pre-menopausal and perimenopausal women were more likely to report a past suicide attempt than post-menopausal women [42]. Furthermore, genome-wide association data [32] highlighted a correlation between suicide attempts and earlier age of menopause. Additionally, one study observed an elevated L genotype frequency in the serotonin transporter (5-HTT) gene in menopausal individuals following a suicide attempt compared to a corresponding fertile group [29].

#### Suicide mortality

The single study presenting data on suicide mortality [38] found that suicide mortality rates were highest in the pre-menopausal group relative to those in the post-menopause.

### Other studies of suicidality

#### Qualitative studies

Two qualitative studies were included. Murphy et al. [47] sought to examine the experiences of midlife in Qatari and Arabic women. In the focus group, a participant discussed the protective influence religion may have against feeling suicidal or engaging in suicidal behaviour. Participants contrasted this protective influence with perceptions of Western communities, where menopausal women might face higher risks of serious mental health challenges or self-harm due to a perceived lack of spiritual or religious beliefs.

Moseley et al. [46] explored perceptions and experiences of the menopause transition in women self-diagnosed or formally diagnosed with autism. Participants mentioned their experience of suicidal ideation in relation to the menopause, with the menopause being described as overwhelming due to the additional stress. Furthermore, participants expressed wanting more research into suicidality and suicide associated with the menopause.

#### Case studies and clinical observations

Data was identified from 11 patients presented in seven case studies and clinical observations [31, 37, 39, 40, 44, 45, 55]. The publications presented or referred to patients who experienced suicidal ideation or thoughts [31, 39, 44], had a suicide attempt [37, 44, 45], or died by suicide [40, 55], all of which were reported to be associated to some extent with the menopause.

#### Quality appraisal

Sixteen studies were appraised for quality using the Quality Appraisal Tool (QATSDD; [28–30, 34, 35, 38, 41–43, 46–48, 51–54]). The remaining 12 studies could not be assessed for quality for the following reasons: case study/clinical observation (n = 7; [31, 37, 39, 40, 44, 45, 55]) conference abstract (n = 3; [33, 49, 50]), poster (n = 1; [36]), not enough description of input data provided (n = 1; [32]). The results of the quality appraisal for the included 16 studies are provided in Table 4 and S4 Table.

**Table 4:** Quality appraisal scores for the included studies.

Ref.	Author	Year	Country	1	2	3	4	5	6	7	8	9	10	11	12	13	14	15	16	Score	%
28	An et al.	2022	South Korea	2	2	2	0	2	3	2	2	1	3	N/A	3	3	N/A	0	3	28/43	66.7
29	Baca-Garcia et al.	2003	Spain	3	3	2	0	1	2	2	2	0	3	N/A	3	0	N/A	0	2	23/43	54.8
30	Baca-Garcia et al.	2010	Spain	2	3	2	1	1	3	2	2	1	3	N/A	3	3	N/A	0	2	28/43	66.7
34	González-Rodríguez et al.	2015a	Spain	2	3	2	0	2	1	0	1	0	3	N/A	3	3	N/A	0	1	21/43	50.0
35	González-Rodríguez et al.	2015b	Spain	2	3	2	0	2	2	1	2	1	3	N/A	3	3	N/A	0	1	25/43	59.5
38	Høyer & Lund	1993	Norway	2	1	1	0	2	2	1	1	0	3	N/A	3	2	N/A	0	1	19/43	45.2
41	Jung et al.	2019	South Korea	2	3	2	0	2	3	0	0	0	3	N/A	3	3	N/A	0	1	22/43	52.4
42	Kornstein et al.	2010	USA	2	3	1	0	3	3	0	3	0	3	N/A	3	3	N/A	0	3	27/43	64.3
43	Lipsitz et al.	2021	Canada	3	3	2	0	1	3	2	2	1	3	N/A	3	3	N/A	0	3	29/43	69.1
46	Moseley et al.	2020	UK	3	3	2	0	1	3	2	2	N/A	N/A	2	N/A	1	2	0	2	23/39	59.0
47	Murphy et al.	2013	Qatar	2	2	3	0	2	2	0	2	N/A	N/A	3	N/A	0	0	0	0	16/39	41.0
48	Nakanishi et al.	2023	Japan	1	3	1	0	2	3	0	3	0	3	N/A	3	3	N/A	0	3	25/43	59.5
51	Ryu et al.	2022	South Korea	1	3	3	0	2	2	2	1	1	3	N/A	3	1	N/A	0	3	25/43	59.5
52	Sherr et al.	2016	UK	2	2	3	0	1	2	1	3	0	3	N/A	3	1	N/A	0	1	22/43	52.4
53	Usall et al.	2009	N/A	1	3	1	0	3	2	0	1	0	3	N/A	3	3	N/A	0	2	22/43	52.4
54	Weiss et al.	2016	USA	2	3	1	0	2	2	2	2	1	3	N/A	3	2	N/A	0	2	25/43	59.5

***Note*.** 12 of the included could not be assessed for quality for the following reasons: case study/clinical observation (n = 7, ([30, 36, 38, 39, 43, 44, 54]) conference abstract (n = 3; [32, 48, 49]), poster (n = 1; [35]), not enough description of input data provided (n = 1; [31]).

Total quality scores ranged from 16 [47] to 29 [43] (*M* = 23.75, SD = 3.47), with percentage quality scores ranging from 41.0% to 69.1%, respectively (M = 57.0%, SD = 7.89). Item 15 (evidence of user involvement in design) was the most poorly scored item, with all studies scoring 0 (i.e., no mention at all). Similarly, item 4 (evidence of sample size considered in terms of analysis) was poorly scored, with all but one study [30] scoring 0 (i.e., no mention at all). Items 10 (fit between research question and method of data collection) and 12 (fit between research question and method of analysis), both of which were only relevant when assessing the quality of quantitative studies, were the most highly rated items, with all included studies scoring 3 (i.e., method of data collection/analysis selected is the most suitable approach to attempt to answer the research question).

## Discussion

The current study aimed to explore the available literature on suicidality in relation to the menopause. Overall, research on this important topic is limited with broad searches only returning just over 2000 studies, of which 28 were deemed relevant to the research question. Unfortunately, there is evidence of poor funding for women’s health research [68, 69], which has contributed to the gaps in healthcare provision for women and a lack of understanding of women’s health conditions. Given that insights into menopausal suicidality can serve as a foundation for more effective and informed menopausal care, including the development of improved strategies for identifying and managing suicidality symptoms, including risk mitigation, increasing funding for women’s health research is crucial.

The studies reviewed revealed a complex relationship between hormonal status and suicidality, with inconsistent findings across various investigations. Broadly considering the relationship between suicidality and menopause, some studies found that menopause and menopause transition was a risk factor for suicidal ideation when compared to the pre-menopause [28, 42, 49], with reports of a statistically significant relationship between suicidal ideation and symptoms of the menopause [50]. Beyond self-report measures of suicidality, studies have found associations between menopause status and severe suicidal intent through hormone level analysis [30], and suicide attempts through genotype analysis [29]. More severe suicidal intent was observed in individuals with low oestrogen and progesterone levels (i.e., due to menopause, amenorrhea, menstruation) after a suicide attempt compared to pre-menopausal individuals in other phases of the menstrual cycle [30]. In addition to low hormonal levels being linked to suicidal behaviour, hormonal sensitivity as determined by genotype was implicated in suicidal attempts with the frequency of the L genotype being higher in suicide attempters in low estradiol states (i.e., the menopause, menstruation [29]).

Conversely, other studies found no association between menopause and suicidal behaviour [35, 41] or suicidal ideation [33, 35, 41, 43, 52, 54], particularly when social support is considered [34]. Moreover, some studies reported rates of suicide mortality were higher in pre-menopausal individuals [38], but it is worth noting that trends in suicide mortality data change over time [4].

Findings were also mixed when examining the relationship between suicidality and the menopause across specific stages. Some studies indicated that the perimenopausal period appears to be a particularly vulnerable time for risk of suicidality, with perimenopausal women exhibiting a higher prevalence of suicidal thoughts compared to both pre-menopausal and post-menopausal women [53], as well as demonstrating a higher likelihood of experiencing suicidal thoughts compared to pre-menopausal women [48]. Conversely, multiple studies identified the post-menopausal phase as a period marked by heightened vulnerability to suicidal thoughts compared to women in the pre-menopause stage [28, 36, 42, 49, 52], women in the perimenopause stage [28, 42] and men [36]. Interestingly, one study also found differences in the reason for suicidal ideation, with women in the post-menopause being more likely to engage in suicidal behaviour to “stop the pain” [36]. Whilst non-significant, this highlights the importance of examining gender and age disaggregated data to comprehensively understand the potential drivers of suicidality unique to specific groups and developing interventions based on those insights. This is particularly important as the same study also reported the post-menopause group is less likely to be deterred from suicidal behaviour [36]. The final group of interest examined in the included studies was early-onset menopause, with menopause onset before the age of 40 (categorised as primary ovarian insufficiency by the study) being associated with a higher risk of suicidal ideation compared to menopause onset after 45 years of age [51]. This association was further corroborated with data from a genome-wide association study identifying a link between an earlier age at menopause and suicide attempts [32]. Genetic studies are certainly valuable at helping improve understanding of the biological mechanisms of menopausal suicidality, however, given the scarcity of genetic studies identified in the current review, further research is needed in order to understand the role genetics may play in determining suicidal behaviour and how this may vary according to hormonal status. Ensuring high quality information regarding hormone status (i.e., pre-menopause, peri-menopause, post-menopause) is included in publicly available genetic datasets will likely facilitate this.

Two studies identified in the current review explored the experience of suicidality in relation to the menopause using qualitative techniques, with data generated from focus groups [45, 46]. These studies provide valuable insights into the diverse experiences of women in their menopause, with one focusing on potentially protective factors of serious mental health symptoms in the menopause such as religion [46] and the other revealing the risks of impaired coping and suicidality during the menopause associated with autism [47]. Qualitative research is essential for understanding the experience of suicidality during the menopause, and is particularly important for capturing the perspectives of groups which may not be typically represented in research. In addition, qualitative research can assist in setting research priorities.

The current review also identified a number of case studies and reporting of clinical experience describing patients with a presentation of severe mental health symptoms and suicidality occurring alongside the menopause. The majority of these case studies presented patients with abrupt onset of suicidality coinciding with onset of the menopause, and reported that the patients had had no such previous psychiatric history. Case studies, whilst not able to provide data on population level incidence and characteristics, can reveal specific circumstances at the individual level which may be associated with suicidality in this phase of life and indicate areas for further research. The earliest of these publications was from 1900 [40], indicating that menopausal suicidality is not a recent phenomenon but, in fact, an experience associated with the menopause which has likely been historically under-recognised and under-reported.

Quality appraisal scores varied, and revealed a consistent lack of involvement in study design with individuals with relevant lived experience. Co-design of research materials is important as it can help ensure the relevancy to the population of interest [70] and ensure vulnerable and hard-to-reach populations are represented to encourage engagement [71]. Additionally, given the sensitive subject matter, involving individuals with relevant lived experience of suicidality, particularly if related to the menopause, would ensure study materials are appropriate and sensitive. In terms of study design, it was deemed that all studies employed appropriate data collection methods relevant to their research question. However, it should be noted that studies that utilised national survey data held some of the largest and richest datasets, allowing for insights into broad spectrums of the population of interest.

Outside of the quality appraisal framework, it is worth noting there are several limitations associated with the studies included in the current review. Firstly, a proportion of the identified studies examined suicidality in relation to the menopause in psychiatric populations [32–36, 39, 42–44, 46, 54, 55] or in populations with physical health conditions (e.g., epilepsy [49] and HIV [52] which are frequently associated with mental health comorbidities [72, 73]. Therefore, such findings will likely not extrapolate to the general population. In this regard, more research in general population samples is warranted to examine the interaction between menopause and suicidality without potential confounding factors due to pre-existing physical and mental health comorbidities.

Additionally, most studies included did not report data on ethnicity. Given that the experience of the menopause varies between ethnic groups [74], it is important that possible variations in suicidality related to the menopause in different ethnic groups are captured.

Further, two studies examining suicide attempts used unspecified past or lifetime suicide attempts as a measure [35, 42] which may reduce their ability to establish the incidence of suicidal behaviour commencing following the onset of menopause. Going forward, studies should aim to more accurately measure whether first episode suicidality occurred prior to, during, or after the menopause transition.

Finally, given variations in the age of onset of menopause [75, 76], using age as a proxy measure of menopause may be flawed. We therefore encourage researchers in all areas of menopause research to utilise other methods of determining menopausal status, and to be transparent in the reporting of these methods.

### Limitations

The findings of the current systematic review should be considered alongside several limitations. Firstly, as the language of included papers was limited to English, there is a risk that relevant literature related to the study focus may have not been included in the final dataset. Additionally, as the decision was made to limit the focus of the current review specifically to the influence of the menopause on suicidality, other confounding variables which may be important in this complex relationship have not been considered. For instance, socioeconomic factors (i.e., unemployment, marital breakdown), mental health diagnoses, and physical health diagnoses have been implicated in suicidality in midlife [3]. Future work should seek to examine additional factors which may mediate or further elucidate the relationship between menopause and suicide. Finally, given the methodological discrepancies between the included studies, including the wide variety of measures of menopause status and suicidality, the current findings present a complex view of the relationship between the menopause and suicidality which is difficult to synthesise. In this regard, further research, particularly in the general population, is warranted to gain a better understanding of the risk of suicidality in this challenging phase of life.

## Conclusions

The evidence on menopause and suicidality is mixed, with conflicting findings on which menopausal stage carries the highest risk. Some studies link hormonal changes to suicidality, but inconsistent results highlight the complexity of this relationship. Historical case studies, qualitative insights, and clinical experiences add depth, showing that menopausal suicidality is not a new issue but one that may have been under-recognized.

This review reveals a lack of research specifically on menopausal suicidality, especially concerning suicide attempts and mortality. Most studies focus on suicidal ideation, leaving gaps in understanding. Future research should prioritize suicide attempts and mortality, using both quantitative and qualitative methods. It’s crucial to standardize definitions of menopause stages and avoid using age as a proxy. Longitudinal studies would help track changes in suicidality across menopause, identifying intervention points. Co-design principles, involving those with lived experiences, should guide research to ensure sensitivity and relevance.

## Supporting information

S1 Table PRISMA checklist. (DOCX)

S2 Table Summary of inclusion decisions. (XLSX)

S3 Table Summary of studies included in the review. (XLSX)

S4 Table Quality assessment: Quality Assessment Tool for Studies with Diverse Designs (QATDSS) scoring. (DOCX)

## References

[1] World Health Organisation. One in 100 deaths is by suicide. 2021 Jun 17 [cited 14 Mar 2024]. Available from: https://www.who.int/news/item/17-06-2021-one-in-100-deaths-is-by-suicide.

[2] Baca-Garcia E, Perez-Rodriguez MM, Mann JJ, Oquendo MA. Suicidal Behavior in Young Women. Psychiatric Clinics of North America. 2008 Jun 1;31(2):317–31. doi: 10.1016/j.psc.2008.01.002 18439451

[3] Qin P, Syeda S, Canetto SS, Arya V, Liu B, Menon V, et al. Midlife suicide: A systematic review and meta-analysis of socioeconomic, psychiatric and physical health risk factors. J Psychiatr Res. 2022 Oct;154:233–41. doi: 10.1016/j.jpsychires.2022.07.037 35961179

[4] Ilic M, Ilic I. Worldwide suicide mortality trends (2000–2019): A joinpoint regression analysis. World J Psychiatry. 2022 Aug 19;12(8):1044–60. doi: 10.5498/wjp.v12.i8.1044 36158305 PMC9476842

[5] Office for National Statistics. Suicides in England and Wales. 2023 Dec 19 [Accessed 14 Mar 2024]. Available from: https://www.ons.gov.uk/peoplepopulationandcommunity/birthsdeathsandmarriages/deaths/bulletins/suicidesintheunitedkingdom/2022registrations

[6] Norwegian Institute of Public Health. Suicide in Norway. 2023 Mar 31 [Accessed 28 Jun 2024]. Available from: https://www.fhi.no/en/he/hin/mental-health/suicide/?term=

[7] Avis NE, Colvin A, Bromberger JT, Hess R, Matthews KA, Ory M, et al. Change in health-related quality of life over the menopausal transition in a multiethnic cohort of middle-aged women: Study of Women’s Health Across the Nation (SWAN). Menopause. 2009;16(5):860–9. 19436224 10.1097/gme.0b013e3181a3cdaf PMC2743857

[8] Vivian-Taylor J, Hickey M. Menopause and depression: Is there a link? Maturitas. 2014 Oct 1;79(2):142–6. doi: 10.1016/j.maturitas.2014.05.014 24951102

[9] Alam MM, Ahmed S, Dipti RK, Siddiquee RE, Hawlader MDH. The prevalence and associated factors of depression during pre-, peri-, and post-menopausal period among the middle-aged women of Dhaka city. Asian Journal of Psychiatry. 2020 Dec 1;54:102312. 32795954 10.1016/j.ajp.2020.102312

[10] Freeman EW. Associations of depression with the transition to menopause. Menopause. 2010 Jul;17(4):823. doi: 10.1097/gme.0b013e3181db9f8b 20531231

[11] Too LS, Spittal MJ, Bugeja L, Reifels L, Butterworth P, Pirkis J. The association between mental disorders and suicide: A systematic review and meta-analysis of record linkage studies. Journal of Affective Disorders. 2019 Dec 1;259:302–13. doi: 10.1016/j.jad.2019.08.054 31450139

[12] Cai H, Xie XM, Zhang Q, Cui X, Lin JX, Sim K, et al. Prevalence of Suicidality in Major Depressive Disorder: A Systematic Review and Meta-Analysis of Comparative Studies. Front Psychiatry. 2021 Sep 16;12. doi: 10.3389/fpsyt.2021.690130 34603096 PMC8481605

[13] Fernandez-Rodrigues V, Sanchez-Carro Y, Lagunas LN, Rico-Uribe LA, Pemau A, Diaz-Carracedo P, et al. Risk factors for suicidal behaviour in late-life depression: A systematic review. World J Psychiatry. 2022 Jan 19;12(1):187–203. doi: 10.5498/wjp.v12.i1.187 35111588 PMC8783161

[14] American Psychiatric Association. Diagnostic and Statistical Manual of mental health disorders. 5th ed. text revision. Arlington, VA: American Psychiatric Association; 2022.

[15] Gibbs Z, Lee S, Kulkarni J. The unique symptom profile of perimenopausal depression. Clinical Psychologist. 2015;19(2):76–84.

[16] Jha MK, Minhajuddin A, Chin Fatt C, Kircanski K, Stringaris A, Leibenluft E, et al. Association between irritability and suicidal ideation in three clinical trials of adults with major depressive disorder. Neuropsychopharmacol. 2020 Dec;45(13):2147–54. doi: 10.1038/s41386-020-0769-x 32663842 PMC7784964

[17] Nappi RE, Kroll R, Siddiqui E, Stoykova B, Rea C, Gemmen E, et al. Global cross-sectional survey of women with vasomotor symptoms associated with menopause: prevalence and quality of life burden. Menopause. 2021 May 24;28(8):875–82. doi: 10.1097/GME.0000000000001793 34033602 PMC8746897

[18] Bromberger JT, Kravitz HM, Chang Y, Randolph JFJ, Avis NE, Gold EB, et al. Does risk for anxiety increase during the menopausal transition? Study of Women’s Health Across the Nation. Menopause. 2013 May;20(5):488. doi: 10.1097/GME.0b013e3182730599 23615639 PMC3641149

[19] Choi HY, Kim SI, Yun KW, Kim YC, Lim WJ, Kim EJ, et al. A Study on Correlation between Anxiety Symptoms and Suicidal Ideation. Psychiatry Investig. 2011 Dec;8(4):320–6. doi: 10.4306/pi.2011.8.4.320 22216041 PMC3246139

[20] Kanwar A, Malik S, Prokop LJ, Sim LA, Feldstein D, Wang Z, et al. The Association Between Anxiety Disorders and Suicidal Behaviors: A Systematic Review and Meta-Analysis. Depression and Anxiety. 2013;30(10):917–29. doi: 10.1002/da.22074 23408488

[21] Bentley KH, Franklin JC, Ribeiro JD, Kleiman EM, Fox KR, Nock MK. Anxiety and its disorders as risk factors for suicidal thoughts and behaviors: A meta-analytic review. Clin Psychol Rev. 2016 Feb;43:30–46. doi: 10.1016/j.cpr.2015.11.008 26688478 PMC4771521

[22] Holman MS, Williams MN. Suicide Risk and Protective Factors: A Network Approach. Archives of Suicide Research. 2022 Jan 2;26(1): 137–154. doi: 10.1080/13811118.2020.1774454 32522102

[23] Martin-Key NA, Funnell EL, Spadaro B, Bahn S. Perceptions of healthcare provision throughout the menopause in the UK: a mixed-methods study. npj Womens Health. 2023 Dec 7;1(1):1–10.

[24] Refaei M, Mardanpour S, Masoumi SZ, Parsa P. Women’s experiences in the transition to menopause: a qualitative research. BMC Women’s Health. 2022 Feb 26;22(1):53. doi: 10.1186/s12905-022-01633-0 35219295 PMC8882304

[25] Moher D, Shamseer L, Clarke M, Ghersi D, Liberati A, Petticrew M, et al. Preferred reporting items for systematic review and meta-analysis protocols (PRISMA-P) 2015 statement. Systematic Reviews. 2015 Jan 1;4(1):1. doi: 10.1186/2046-4053-4-1 25554246 PMC4320440

[26] Cooke A, Smith D, Booth A. Beyond PICO: The SPIDER Tool for Qualitative Evidence Synthesis. Qual Health Res. 2012 Oct 1;22(10):1435–43. doi: 10.1177/1049732312452938 22829486

[27] Sirriyeh R, Lawton R, Gardner P, Armitage G. Reviewing studies with diverse designs: the development and evaluation of a new tool. J Eval Clin Pract. 2012 Aug;18(4):746–52. doi: 10.1111/j.1365-2753.2011.01662.x 21410846

[28] An SY, Kim Y, Kwon R, young Lim G, Choi HR, Namgoung S, et al. Depressive symptoms and suicidality by menopausal stages among middle-aged Korean women. Epidemiology and Psychiatric Sciences. 2022 Jan;31:e60. doi: 10.1017/S2045796022000439 36017644 PMC9428901

[29] Baca-Garcia E, Vaquero C, Diaz-Sastre C, Ceverino A, Saiz-Ruiz J, Fernández-Piquera J, et al. A pilot study on a gene-hormone interaction in female suicide attempts. Eur Arch Psychiatry Clin Neurosci. 2003 Dec;253(6):281–5. doi: 10.1007/s00406-003-0441-6 14714116

[30] Baca-Garcia E, Diaz-Sastre C, Ceverino A, Perez-Rodriguez MM, Navarro-Jimenez R, Lopez-Castroman J, et al. Suicide attempts among women during low estradiol/low progesterone states. Journal of Psychiatric Research. 2010 Mar 1;44(4):209–14. doi: 10.1016/j.jpsychires.2009.08.004 19782376

[31] Fleming JAE, Warneboldt RB. Multiple Sleep Pathologies Presenting as Depression. Can Fam Physician. 1990 Jun;36:1185–9. 21233990 PMC2280489

[32] Fujikane D, Ohi K, Kuramitsu A, Takai K, Muto Y, Sugiyama S, et al. Genetic correlations between suicide attempts and psychiatric and intermediate phenotypes adjusting for mental disorders. Psychol Med. 2024 Feb;54(3):488–94. doi: 10.1017/S0033291723002015 37559484

[33] González-Rodríguez A, Molina-Andreu O, Imaz Gurrutxaga ML, Penadés Rubio R, Bernardo Arroyo M, Catalán Campos R. Oestrogen related features and gynaecological service use in delusional disorder. European Psychiatry. 2013 Jan 1;28:1.21920709

[34] González-Rodríguez A, Molina-Andreu O, Penadés Rubio R, Catalán Campos R, Bernardo Arroyo M. Reproductive variables and gynaecological service use in delusional disorder outpatients. Journal of psychiatry and mental health. 2015 Apr 1;8(2):92–6. 10.1016/j.rpsm.2013.10.001 24378254

[35] González-Rodríguez A, Molina-Andreu O, Penadés R, Garriga M, Pons A, Catalán R, et al. Delusional Disorder over the Reproductive Life Span: The Potential Influence of Menopause on the Clinical Course. Schizophrenia Research and Treatment. 2015 Oct 27;2015:e979605. doi: 10.1155/2015/979605 26600949 PMC4639664

[36] González-Rodríguez A, Catalan R, Penades R, Ruiz Cortes V, Torra M, Bernardo M. Suicidal ideation and suicidal behaviour in postmenopausal schizophrenia women: the relevance of suicide risk assessment. Climacteric. Abstracts for 15th World Congress on Menopause. 2016 Sep 26;19:76–77.

[37] Hemphill RE, Reiss M. Corticotrophic Hormone for Pituitary Cachexia. Br Med J. 1944 Aug 12;2(4362):211–3. doi: 10.1136/bmj.2.4362.211 20785591 PMC2285999

[38] Høyer G, Lund E. Suicide among women related to number of children in marriage. Arch Gen Psychiatry. 1993 Feb;50(2):134–7. doi: 10.1001/archpsyc.1993.01820140060006 8427553

[39] Incalzi RA, Gemma A, Capparella O. Sleep apnoea syndrome—a too frequent misdiagnosis. Postgrad Med J. 1989 May;65(763):345. doi: 10.1136/pgmj.65.763.345 2608575 PMC2429300

[40] Jones HM. Affections of the Female Genitalia as Causal Factors in the Etiology of Neuroses and Insanity, and Their Special Bearing on the Operative Treatment of the Insane. Edinb Med J. 1900 Oct;8(4):305–22.

[41] Jung M, Koo H, Noh JH. Association Between Depression And Early Menopause In South Korean Women. The Internet Journal of Gynecology and Obstetrics. 2019 Aug 20;23(1).

[42] Kornstein SG, Young EA, Harvey AT, et al. The influence of menopause status and postmenopausal use of hormone therapy on presentation of major depression in women. Menopause. 2010 Jul;17(4):828–839. doi: 10.1097/gme.0b013e3181d770a8 20616669 PMC2949279

[43] Lipsitz O, McIntyre RS, Rodrigues NB, Lee Y, Cha DS, Gill H, et al. Intravenous ketamine for postmenopausal women with treatment-resistant depression: Results from the Canadian Rapid Treatment Center of Excellence. J Psychiatr Res. 2021 Apr;136:444–51. doi: 10.1016/j.jpsychires.2020.08.002 32948309

[44] Little GA, Cameron DE. The Effects of Theelin on Anxiety. Can Med Assoc J. 1937 Aug;37(2):144–50. 20320703 PMC1562185

[45] Zhiqiang L, Bin S, Min F, Yufang L. Leiomyosarcoma of cervical stump following subtotal hysterectomy: a case report and review of literature. Eur J Gynaecol Oncol. 2016;37(1):148–51. 27048131

[46] Moseley RL, Druce T, Turner-Cobb JM. ‘When my autism broke’: A qualitative study spotlighting autistic voices on menopause. Autism. 2020 Aug;24(6):1423–37. doi: 10.1177/1362361319901184 32003226 PMC7376624

[47] Murphy MM, Verjee MA, Bener A, Gerber LM. The hopeless age? A qualitative exploration of the experience of menopause in Arab women in Qatar. Climacteric. 2013 Oct;16(5):550–4. doi: 10.3109/13697137.2013.771330 23374139

[48] Nakanishi M, Endo K, Yamasaki S, Stanyon D, Sullivan S, Yamaguchi S, et al. Association between menopause and suicidal ideation in mothers of adolescents: A longitudinal study using data from a population-based cohort. J Affect Disord. 2023 Nov 1;340:529–34. doi: 10.1016/j.jad.2023.08.055 37573891

[49] Patel S, Foldvary-Schaefer N, Jehi L, Tesar G, Viguera A. Are Menopausal Women with Epilepsy at Higher Risk for Mood Disorders and Suicide Risk?. Neurology. 2012 Apr 23;78.

[50] Rocha JPB, Rocha MCB, Brito MFS, Pinho L, Rocha MS, Souza SJ, et al. Prevalence of suicidal ideation and associated factors in climacteric women. Menopause. 30th Annual Meeting of The North America Menopause Society September 25–28, 2019, Chicago, IL. Menopause. 2019 Dec;26(12):1445.

[51] Ryu KJ, Park H, Jeong Y, Nam S, Jeong HG, Kim T. Age at Menopause and Suicidal Ideation in Menopausal Women: A Study of Korea National Health and Nutrition Examination Survey Data. Journal of Korean Medical Science. 2022 Nov 21;37(45):e330. doi: 10.3346/jkms.2022.37.e330 36413799 PMC9678656

[52] Sherr L, Molloy A, Macedo A, Croome N, Johnson MA. Ageing and menopause considerations for women with HIV in the UK. J Virus Erad. 2016 Oct 5;2(4):215–8. 27781103 10.1016/S2055-6640(20)30874-8 PMC5075348

[53] Usall J, Pinto-Meza A, Fernández A, de Graaf R, Demyttenaere K, Alonso J, et al. Suicide ideation across reproductive life cycle of women. Results from a European epidemiological study. J Affect Disord. 2009 Jul;116(1–2):144–7. doi: 10.1016/j.jad.2008.12.006 19155069

[54] Weiss SJ, Simeonova DI, Kimmel MC, Battle CL, Maki PM, Flynn HA. Anxiety and physical health problems increase the odds of women having more severe symptoms of depression. Arch Womens Ment Health. 2016 Jun;19(3):491–9. doi: 10.1007/s00737-015-0575-3 26403982

[55] Worsley A, Smythe J, Kulkarni J. A case of perimenopausal depression. Aust N Z J Psychiatry. 2016 Nov 1;50(11):1109–1109. doi: 10.1177/0004867416671597 27698111

[56] Harlow SD, Gass M, Hall JE, Lobo R, Maki P, Rebar RW, et al. Executive summary of the Stages of Reproductive Aging Workshop + 10: addressing the unfinished agenda of staging reproductive aging. Menopause. 2012 Apr;19(4):387–95. doi: 10.1097/gme.0b013e31824d8f40 22343510 PMC3340903

[57] International Menopause Society. Menopause Terminology. [Accessed 2024 Mar 15]. Available from: https://www.imsociety.org/education/menopause-terminology/

[58] Posner K, Brown GK, Stanley B, Brent DA, Yershova KV, Oquendo MA, et al. The Columbia–Suicide Severity Rating Scale: Initial Validity and Internal Consistency Findings From Three Multisite Studies With Adolescents and Adults. AJP. 2011 Dec;168(12):1266–77. doi: 10.1176/appi.ajp.2011.10111704 22193671 PMC3893686

[59] Rush AJ, Trivedi MH, Ibrahim HM, Carmody TJ, Arnow B, Klein DN, et al. The 16-Item Quick Inventory of Depressive Symptomatology (QIDS), clinician rating (QIDS-C), and self-report (QIDS-SR): a psychometric evaluation in patients with chronic major depression. Biol Psychiatry. 2003 Sep 1;54(5):573–83. doi: 10.1016/s0006-3223(02)01866-8 12946886

[60] Trivedi MH, Rush AJ, Ibrahim HM, Carmody TJ, Biggs MM, Suppes T, et al. The Inventory of Depressive Symptomatology, Clinician Rating (IDS-C) and Self-Report (IDS-SR), and the Quick Inventory of Depressive Symptomatology, Clinician Rating (QIDS-C) and Self-Report (QIDS-SR) in public sector patients with mood disorders: a psychometric evaluation. Psychol Med. 2004 Jan;34(1):73–82. doi: 10.1017/s0033291703001107 14971628

[61] Sterling M. General Health Questionnaire—28 (GHQ-28). J Physiother. 2011;57(4):259. doi: 10.1016/S1836-9553(11)70060-1 22093128

[62] Kroenke K, Spitzer RL, Williams JBW. The PHQ-9. J Gen Intern Med. 2001 Sep;16(9):606–13. 11556941 10.1046/j.1525-1497.2001.016009606.x PMC1495268

[63] Beck AT, Kovacs M, Weissman A. Assessment of suicidal intention: the Scale for Suicide Ideation. J Consult Clin Psychol. 1979 Apr;47(2):343–52. doi: 10.1037//0022-006x.47.2.343 469082

[64] Harriss L, Hawton K. Suicidal intent in deliberate self-harm and the risk of suicide: the predictive power of the Suicide Intent Scale. J Affect Disord. 2005 Jun;86(2–3):225–33. doi: 10.1016/j.jad.2005.02.009 15935242

[65] Sheehan DV, Lecrubier Y, Sheehan KH, Amorim P, Janavs J, Weiller E, et al. The Mini-International Neuropsychiatric Interview (M.I.N.I.): the development and validation of a structured diagnostic psychiatric interview for DSM-IV and ICD-10. J Clin Psychiatry. 1998;59 Suppl 20:22–33;quiz 34–57. 9881538

[66] National Institutes of Health (NIH). [Accessed 2024 Mar 15]. National Institutes of Health (NIH). Available from: https://www.nih.gov/

[67] Moriyama IM. The eighth revision of the International Classification of Diseases. Am J Public Health Nations Health. 1966 Aug;56(8):1277–80. doi: 10.2105/ajph.56.8.1277 5950717 PMC1257239

[68] McKinsey. Closing the women’s health gap. 2024 Jan 17 [Accessed 2024 Mar 15]. Available from: https://www.mckinsey.com/mhi/our-insights/closing-the-womens-health-gap-a-1-trillion-dollar-opportunity-to-improve-lives-and-economies

[69] Women’s health: end the disparity in funding. Nature. 2023 May 3;617(7959):8–8. 37138115 10.1038/d41586-023-01472-5

[70] Slattery P, Saeri AK, Bragge P. Research co-design in health: a rapid overview of reviews. Health Research Policy and Systems. 2020 Feb 11;18(1):17. doi: 10.1186/s12961-020-0528-9 32046728 PMC7014755

[71] Moll S, Wyndham-West M, Mulvale G, Park S, Buettgen A, Phoenix M, et al. Are you really doing “codesign”? Critical reflections when working with vulnerable populations. BMJ Open. 2020 Nov 3;10(11):e038339. doi: 10.1136/bmjopen-2020-038339 33148733 PMC7640510

[72] Jones R, Rickards H, Cavanna AE. The prevalence of psychiatric disorders in epilepsy: a critical review of the evidence. Functional Neurology. 2010;25(4):191–4. 21388578

[73] Orza L, Bewley S, Logie CH, Crone ET, Moroz S, Strachan S, et al. How does living with HIV impact on women’s mental health? Voices from a global survey. Journal of the International AIDS Society. 2015;18(6S5):20289. doi: 10.7448/IAS.18.6.20289 26643460 PMC4672402

[74] Green R, Santoro N. Menopausal Symptoms and Ethnicity: The Study of Women’s Health across the Nation. Womens Health (Lond Engl). 2009 Mar 1;5(2):127–33. doi: 10.2217/17455057.5.2.127 19245351 PMC3270699

[75] Palacios S, Henderson VW, Siseles N, Tan D, Villaseca P. Age of menopause and impact of climacteric symptoms by geographical region. Climacteric. 2010 Oct;13(5):419–28. doi: 10.3109/13697137.2010.507886 20690868

[76] Ceylan B, Özerdoğan N. Factors affecting age of onset of menopause and determination of quality of life in menopause. Turk J Obstet Gynecol. 2015 Mar;12(1):43–9. doi: 10.4274/tjod.79836 28913040 PMC5558404

