# Supplementary material for: Examining suicidality in relation to the menopause: A systematic review
Source: PLOS Ment Health. 2024 Nov 13;1(6):e0000161. doi: 10.1371/journal.pmen.0000161 (PMC12798595; doi:10.1371/journal.pmen.0000161)
Supplement: S4 Table — (DOCX) [file pmen.0000161.s004.docx]

**S4 Table.** **Quality assessment: Quality Assessment Tool for Studies with Diverse Designs (QATDSS) scoring**

| **Item** | **Criteria** | **0 = not at all** | **1= very slightly** | **2 = moderately** | **3= complete** |
| --- | --- | --- | --- | --- | --- |
| **1** | Explicit theoretical framework | No mention at all | Reference to broad theoretical basis | Reference to a specific theoretical basis | Explicit statement of theoretical framework and/or constructs applied to the research |
| **2** | Statement of aims/objectives in main report | No mention at all | General reference to aim/objective at some point in the report including abstract | Reference to broad aims/objectives in main body of report | Explicit statement of aims/objectives in main body of report |
| **3** | Clear description of research setting | No mention at all | General description of research area and background, e.g. ‘in primary care’ | General description of research problem in the target population, e.g. ‘among GPs in primary care’ | Specific description of the research problem and target population in the context of the study, e.g. nurses and doctors from GP practices in the east midlands |
| **4** | Evidence of sample size considered in terms of analysis | No mention at all | Basic explanation for choice of sample size. Evidence that size of the sample has been considered in the study design | Evidence of consideration of sample size in terms of saturation/information redundancy or to fit generic analytical requirements | Explicit statement of data being gathered until information redundancy/saturation was reached to fit exact calculations for analytical requirements |
| **5** | Representative sample of target group of a reasonable size | No statement of target group | Sample is limited but represents some of the target group or representative but very small | Sample is somewhat diverse but not entirely representative, for example, inclusive of all age groups, experience but only one workplace. Requires discussion of target population to determine what sample is required to be representative | Sample includes individuals to represent a cross section of the target population, considering factors such as experience, age and workplace |
| **6** | Description of procedure for data collection | No mention at all | Very basic and brief outline of data collection procedure, e.g. ‘using a questionnaire distributed to staff’ | States each stage of data collection procedure but with limited detail, or states some stages in details but omits others | Detailed description of each stage of the data collection procedure, including when, where and how data were gathered |
| **7** | Rationale for choice of data collection tool(s) | No mention at all | Very limited explanation for choice of data collection tool(s) | Basic explanation of rationale for choice of data collection tool(s), e.g. based on use in a prior similar study | Detailed explanation of rationale for choice of data collection tool(s), e.g. relevance to the study aims and assessments of tool quality either statistically, e.g. for reliability & validity, or relevant qualitative assessment |
| **8** | Detailed recruitment data | No mention at all | Minimal recruitment data, e.g. no. of questionnaire sent and no. returned | Some recruitment information but not complete account of the recruitment process, e.g. recruitment figures but no information on strategy used | Complete data regarding no. approached, no. recruited, attrition data where relevant, method of recruitment |
| **9** | Statistical assessment of reliability and validity of measurement tool(s) (Quantitative studies only) | No mention at all | Reliability and validity of measurement tool(s) discussed, but not statistically assessed | Some attempt to assess reliability and validity of measurement tool(s) but insufficient, e.g. attempt to establish test–retest reliability is unsuccessful but no action is taken | Suitable and thorough statistical assessment of reliability and validity of measurement tool(s) with reference to the quality of evidence as a result of the measures used |
| **10** | Fit between research question and method of data collection (Quantitative studies only) | No research question stated | Method of data collection can only address some aspects of the research question | Method of data collection can address the research question but there is a more suitable alternative that could have been used or used in addition | Method of data collection selected is the most suitable approach to attempt answer the research question |
| **11** | Fit between research question and format and content of data collection tool e.g. interview schedule (Qualitative studies only) | No research question stated | Structure and/or content only suitable to address the research question in some aspects or superficially | Structure & content allows for data to be gathered broadly addressing the stated research question(s) but could benefit from greater detail | Structure & content allows for detailed data to be gathered around all relevant issues required to address the stated research question(s) |
| **12** | Fit between research question and method of analysis (Quantitative studies only) | No mention at all | Method of analysis can only address the research question basically or broadly | Method of analysis can address the research question but there is a more suitable alternative that could have been used or used in addition to offer greater detail | Method of analysis selected is the most suitable approach to attempt answer the research question in detail, e.g. for qualitative IPA preferable for experiences vs. content analysis to elicit frequency of occurrence of events, etc |
| **13** | Good justification for analytic method selected | No mention at all | Basic explanation for choice of analytical method | Fairly detailed explanation of choice of analytical method | Detailed explanation for choice of analytical method based on nature of research question(s) |
| **14** | Assessment of reliability of analytic process (Qualitative studies only) | No mention at all | More than one researcher involved in the analytical process but no further reliability assessment | Limited attempt to assess reliability, e.g. reliance on one method | Use of a range of methods to assess reliability, e.g. triangulation, multiple researchers, varying research backgrounds |
| **15** | Evidence of user involvement in design | No mention at all | Use of pilot study but no involvement in planning stages of study design | Pilot study with feedback from users informing changes to the design | Explicit consultation with steering group or statement or formal consultation with users in planning of study design |
| **16** | Strengths and limitations critically discussed | No mention at all | Very limited mention of strengths and limitations with omissions of many key issues | Discussion of some of the key strengths and weaknesses of the study but not complete | Discussion of strengths and limitations of all aspects of study including design, measures, procedure, sample & analysis |

Scoring: 0=Not at all; 1=Very slightly; 2= Moderately; 3=Completely.

Sirriyeh R, Lawton R, Gardner P, Armitage G. Reviewing studies with diverse designs: the development and evaluation of a new tool. J Eval Clin Pract. 2012 Aug;18(4):746–52.

| **Title** | **Author** | **Year** | **Country** | **1** | **2** | **3** | **4** | **5** | **6** | **7** | **8** | **9** | **10** | **11** | **12** | **13** | **14** | **15** | **16** | **Score** | **%** |
| --- | --- | --- | --- | --- | --- | --- | --- | --- | --- | --- | --- | --- | --- | --- | --- | --- | --- | --- | --- | --- | --- |
| Age at Menopause and Suicidal Ideation in Menopausal Women: A Study of Korea National Health and Nutrition Examination Survey Data | Ryu et al. | 2022 | South Korea | 1 | 3 | 3 | 0 | 2 | 2 | 2 | 1 | 1 | 3 | N/A | 3 | 1 | N/A | 0 | 3 | 25/43 | 59.52 |
| Ageing and menopause considerations for women with HIV in the UK | Sherr et al. | 2016 | UK | 2 | 2 | 3 | 0 | 1 | 2 | 1 | 3 | 0 | 3 | N/A | 3 | 1 | N/A | 0 | 1 | 22/43 | 52.38 |
| Anxiety and physical health problems increase the odds of women having more severe symptoms of depression | Weiss et al. | 2016 | USA | 2 | 3 | 1 | 0 | 2 | 2 | 2 | 2 | 1 | 3 | N/A | 3 | 2 | N/A | 0 | 2 | 25/43 | 59.52 |
| The association between abortion experience and postmenopausal suicidal ideation and mental health: Results from the 5th Korean National Health and Nutrition Examination Survey (KNHANES V) | Wie et al. | 2019 | South Korea | 2 | 2 | 2 | 0 | 2 | 3 | 0 | 0 | 0 | 3 | N/A | 3 | 2 | N/A | 0 | 3 | 22/43 | 52.38 |
| Association between depression and early menopause in South Korean women | Jung et al. | 2019 | South Korea | 2 | 3 | 2 | 0 | 2 | 3 | 0 | 0 | 0 | 3 | N/A | 3 | 3 | N/A | 0 | 1 | 22/43 | 52.38 |
| Association between menopause and suicidal ideation in mothers of adolescents: A longitudinal study using data from a population-based cohort | Nakanishi et al. | 2023 | Japan | 1 | 3 | 1 | 0 | 2 | 3 | 0 | 3 | 0 | 3 | N/A | 3 | 3 | N/A | 0 | 3 | 25/43 | 59.52 |
| Causes of death among women with breast cancer: A follow-up study of 50 481 women with breast cancer in Finland | Katuwal et al. | 2021 | Finland | 2 | 2 | 1 | 0 | 3 | 3 | 0 | 2 | 0 | 3 | N/A | 3 | 3 | N/A | 0 | 2 | 24/43 | 57.14 |
| Delusional Disorder over the Reproductive Life Span: The Potential Influence of Menopause on the Clinical Course | González-Rodríguez et al. | 2015 | Spain | 2 | 3 | 2 | 0 | 2 | 2 | 1 | 2 | 1 | 3 | N/A | 3 | 3 | N/A | 0 | 1 | 25/43 | 59.52 |
| Depressive symptoms and suicidality by menopausal stages among middle-aged Korean women | An et al. | 2022 | South Korea | 2 | 2 | 2 | 0 | 2 | 3 | 2 | 2 | 1 | 3 | N/A | 3 | 3 | N/A | 0 | 3 | 28/43 | 66.67 |
| Factors affecting suicidal ideation among premenopausal and postmenopausal women | Kim et al. | 2020 | South Korea | 3 | 3 | 2 | 0 | 2 | 3 | 2 | 2 | 1 | 3 | N/A | 3 | 3 | N/A | 0 | 2 | 29/43 | 69.05 |
| Health characteristics of postmenopausal women with breast implants | Rubin et al. | 2010 | USA | 1 | 2 | 3 | 0 | 3 | 2 | 1 | 2 | 1 | 3 | N/A | 2 | 2 | N/A | 0 | 1 | 23/43 | 54.76 |
| The hopeless age? A qualitative exploration of the experience of menopause in Arab women in Qatar | Murphy et al. | 2013 | Qatar | 2 | 2 | 3 | 0 | 2 | 2 | 0 | 2 | N/A | N/A | 3 | N/A | 0 | 0 | 0 | 0 | 16/39 | 41.03 |
| Increased suicide risk among patients oophorectomized following benign conditions and its association with comorbidities | Chiu et al. | 2020 | Taiwan | 3 | 3 | 2 | 0 | 3 | 3 | 0 | 3 | 0 | 3 | N/A | 3 | 3 | N/A | 0 | 2 | 28/43 | 66.67 |
| The influence of menopause status and postmenopausal use of hormone therapy on presentation of major depression in women | Kornstein et al. | 2010 | USA | 2 | 3 | 1 | 0 | 3 | 3 | 0 | 3 | 0 | 3 | N/A | 3 | 3 | N/A | 0 | 3 | 27/43 | 64.29 |
| Intravenous ketamine for postmenopausal women with treatment-resistant depression: Results from the Canadian Rapid Treatment Center of Excellence | Lipsitz et al. | 2021 | Canada | 3 | 3 | 2 | 0 | 1 | 3 | 2 | 2 | 1 | 3 | N/A | 3 | 3 | N/A | 0 | 3 | 29/43 | 69.05 |
| A pilot study on a gene-hormone interaction in female suicide attempts | Baca-Garcia et al. | 2003 | Spain | 3 | 3 | 2 | 0 | 1 | 2 | 2 | 2 | 0 | 3 | N/A | 3 | 0 | N/A | 0 | 2 | 23/43 | 54.76 |
| Suicide among women related to number of children in marriage | Høyer & Lund | 1993 | Norway | 2 | 1 | 1 | 0 | 2 | 2 | 1 | 1 | 0 | 3 | N/A | 3 | 2 | N/A | 0 | 1 | 19/43 | 45.24 |
| Suicide attempts among women during low estradiol/low progesterone states | Baca-Garcia et al. | 2010 | Spain | 2 | 3 | 2 | 1 | 1 | 3 | 2 | 2 | 1 | 3 | N/A | 3 | 3 | N/A | 0 | 2 | 28/43 | 66.67 |
| Suicide ideation across reproductive life cycle of women. Results from a European epidemiological study | Usall et al. | 2009 | N/A | 1 | 3 | 1 | 0 | 3 | 2 | 0 | 1 | 0 | 3 | N/A | 3 | 3 | N/A | 0 | 2 | 22/43 | 52.38 |
| When my autism broke': A qualitative study spotlighting autistic voices on menopause | Moseley et al. | 2020 | UK | 3 | 3 | 2 | 0 | 1 | 3 | 2 | 2 | N/A | N/A | 2 | N/A | 1 | 2 | 0 | 2 | 23/39 | 58.97 |
| [Reproductive variables and gynaecological service use in delusional disorder outpatients] | Gonzalez-Rodriguez et al. | 2013 | Spain | 2 | 3 | 2 | 0 | 2 | 1 | 0 | 1 | 0 | 3 | N/A | 3 | 3 | N/A | 0 | 1 | 21/43 | 50.00 |
| Noncontraceptive estrogens and mortality: long-term follow-up of women in the Walnut Creek Study | Petitti et al. | 1987 | USA | 2 | 0 | 3 | 0 | 3 | 3 | 0 | 2 | 0 | 3 | N/A | 2 | 2 | N/A | 0 | 2 | 22/43 | 52.38 |
